# Serum zinc deficiency is a potential risk factor for the occurrence of levodopa-induced dyskinesia in drug-naïve Parkinson’s disease

**DOI:** 10.3389/fnagi.2023.1282367

**Published:** 2023-11-02

**Authors:** Joung Eun Kim, Hyo Sang Lee, Wooyoung Jang

**Affiliations:** ^1^Department of Family Medicine, Asan Medical Center, University of Ulsan College of Medicine, Seoul, Republic of Korea; ^2^Department of Nuclear Medicine, Gangneung Asan Hospital, University of Ulsan College of Medicine, Gangneung, Republic of Korea; ^3^Department of Neurology, Gangneung Asan Hospital, University of Ulsan College of Medicine, Gangneung, Republic of Korea

**Keywords:** Parkinson’s disease, levodopa-induced dyskinesia, zinc, heavy metal, risk factors

## Abstract

**Background:**

Since environmental factors, especially heavy metals, were highlighted in the pathogenesis of Parkinson’s disease (PD), there are many epidemiologic studies regarding heavy metals and PD risk. However, longitudinal studies regarding the impacts of heavy metals on motor and nonmotor symptoms of PD are scarce.

**Methods:**

In the current study, we compared the serum levels of five heavy metals, such as zinc(Zn), copper(Cu), lead(Pb), mercury(Hg), and manganese(Mn), in 111 previously drug-naïve PD patients (*n* = 111) retrospectively. Among these 111 patients, 65 were PD patients without levodopa-induced dyskinesia (LID), while the other 46 had LID. We assembled clinical characteristics of PD and performed correlation analysis with heavy metal levels. At baseline, all subjects were examined with ^18^F-N-(3-fluoropropyl)-2β-carboxymethoxy-3β-(4-iodophenyl) nortropane positron emission tomography/computed tomography (FP-CIT PET/CT). We used Cox proportional hazards regression analysis for determining factors relevant to the time to LID development in PD subjects.

**Results:**

Zn deficiency was significantly higher in the PD with LID group than in the PD without LID group (79.58 ± 12.28 versus 88.16 ± 15.15 μg/L). Lower serum Zn levels were significantly correlated with age of onset, levodopa equivalent daily dose (LEDD) at 3 months, and Korean version of the Mini-Mental State Examination (K-MMSE) scores (*r* = 0.16, *p* < 0.05, *r* = − 0.20, *p* < 0.01, *r* = 0.28, *p* < 0.01). Additionally, Zn deficiency was associated with a reduced time to LID development in the adjusted model (HR 0.978, 95% CI 0.956–0.999).

**Conclusion:**

This study suggests that serum Zn deficiency might be a risk factor for LID in drug-naïve PD patients.

## Introduction

L-3,4-dihydroxyphenylalanine (L-DOPA) is currently the best choice for managing Parkinson’s disease (PD) and the most effective option for ameliorating motor symptoms of PD and maintaining quality of life (Thanvi et al., 2007; Connolly and Lang, 2014). However, long-term application of L-DOPA is known to be associated with various motor complications, such as motor fluctuation and dyskinesia (Ahlskog and Muenter, 2001; Matsuyama et al., 2021). Levodopa-induced dyskinesia (LID) consists of involuntary movement such as choreic movement, dystonia, or a combination of various movement phenomena; this complication can be disabling and worsen quality of life in PD patients (Thanvi et al., 2007).

The Olmsted County cohort revealed that the overall LID frequency at any severity was 30% at 5 years and increased to 59% after 10 years of L-DOPA treatment (Van Gerpen et al., 2006). Ahlskog and Muenter (2001) also reported a 4- to 6-year LID risk of 36% in a systematic review. Regarding risk factors for the development of LID, a young age at onset, a long duration of treatment, a high levodopa dosage, female gender, and low body weight have been proposed. Furthermore, some reports, albeit contested by other studies, show that genetic variants and certain features shown on dopamine transport imaging may contribute to the development of LID.

Although the mechanism of LID remains inconclusive, nigral cell degeneration and the pharmacokinetic characteristics of L-DOPA treatment as chronic pulsatile stimulation could result in functional disorder in basal ganglia circuits, which leads to the occurrence of LID (Jenner, 2008). Furthermore, much recent evidence suggests contributions by nondopaminergic systems such as the glutaminergic and opioid systems, A2A adenosine receptors and molecular epigenetic changes (Sikora and Ouagazzal, 2021; Bandopadhyay et al., 2022). However, much evidence emphasizes that nigrostriatal degeneration is essential for the development of LID (Jeong et al., 2018; Yoo et al., 2018; Jeong et al., 2022). Di Monte et al. compared the thresholds of nigrostriatal damage necessary to produce parkinsonism and LID in squirrel monkeys lesioned with 1-methyl-4-phenyl-1,2,3,6-tetrahydropyridine (MPTP). They reported that moderate nigrostriatal impairment that does not induce clinical parkinsonism still poses a risk of LID (Di Monte et al., 2000). Additionally, Hong et al. (2014) revealed through a retrospective study conducted with 127 *de novo* PD patients who completed ^18^F-N-(3-fluoropropyl)-2β-carboxymethoxy-3β-(4-iodophenyl) nortropane positron emission tomography/computed tomography (FP-CIT PET/CT) that the rates of dopamine uptake by the dopamine transporter (DAT) in the anterior, posterior, and whole putamen were significant predictors of the development of LID. Therefore, factors that could be detrimental to the nigrostriatal system or responsible for its neurodegeneration could be associated with the development of LID in PD.

Although the exact mechanism of neurodegeneration in PD remains inconclusive, the role of environmental factors in PD pathogenesis has gained attention (Lai et al., 2002). In particular, heavy metals are perceived to cause free radical formation through the Fenton-Haber-Weiss reaction (Ball et al., 2019). These reactive oxygen species (ROS) cause oxidative stress, damaging function of mitochondria, while inducing deoxyribonucleic acid (DNA) fragmentation and protein misfolding, which in the end leads to neurodegeneration (Al-Harthi et al., 2022; Vellingiri et al., 2022; Lee et al., 2023). Furthermore, through numerous epidemiological studies, significant relationships between PD and long-term exposure to heavy metals such as mercury (Hb), lead (Pb), manganese (Mn), copper (Cu), and zinc (Zn) have been reported (Tchounwou et al., 2012; Raj et al., 2021; Vellingiri et al., 2022).

In contrast, some heavy metals, such as Mn, iron (Fe), Cu, and Zn, are considered essential trace elements that function as cofactors for many enzymes, and the disruption of heavy metal homeostasis can also result in detrimental effects on the neurological system (Fraga, 2005). For instance, Uversky and Fink mentioned that low concentrations of some metal ions could directly result in α-synuclein (α-syn) fibril formation, which is involved in essential pathologic process in PD (Uversky et al., 2001), and Atarod et al. (2022) also reported that different metal cations could also accelerate α-syn fibril formation and its structural changes with various degrees of cytotoxicity. Therefore, it could be postulated that the dyshomeostasis of heavy metals is an important environmental factor for the neurodegenerative process in PD (Tomita and Yoshikawa, 2002).

Although many epidemiologic studies have been conducted regarding heavy metals and PD risk, longitudinal studies on motor and nonmotor symptoms of PD are rare. In a prior study, we revealed that Zn affects PD dementia conversion and that heavy metal levels could possibly affect the progression of PD (Lee et al., 2023). Considering that one of the prerequisites of LID is degeneration of the nigrostriatal system and that heavy metals take part in neurodegeneration, it can be hypothesized that heavy metals in *de novo* PD patients are related to LID since they can affect the progression of neurodegeneration.

Thus, we compared five heavy metals (Zn, Cu, Pb, Hg, and Mn), retrospectively between the group with LID occurrence and the group without LID occurrence in drug naïve PD. Additionally, we tried to determine whether serum heavy metal levels at the time of diagnosis have an influence on the time to LID occurrence.

## Materials and methods

### Patients and clinical assessment

We conducted a retrospective study involving 111 drug-naïve PD patients who attended the Gangneung Asan Hospital Movement Disorder Clinic between January 2011 and November 2020. The inclusion criteria for PD diagnosis were based on the Movement Disorder Society clinical diagnostic criteria for PD (Postuma et al., 2015). Additionally, FP- CIT PET/CT was examined for all patients to confirm the presence of relevant findings. Following their initial diagnosis, all participants were longitudinally followed for at least 30 months. During this period, they attended outpatient clinics every 2–3 months for regular assessments. The assessments primarily included comprehensive neurological examinations and a detailed history-taking process by a movement disorder specialist. The following is in the exclusion criteria: (1) prior or current intake of levodopa medication; (2) modified Hoehn and Yahr (H&Y) stage more or equivalent to 2.5, as well as showing advanced PD symptoms such as dementia or dysphagia; (3) comorbidities that could affect severity of PD such as cerebrovascular disease, depression, endocrine disease, normal pressure hydrocephalus, alcohol overuse; (4) severe white matter alteration; (5) possible multiple system atrophy, progressive supranuclear palsy, and corticobasal syndrome; and (6) possibility of secondary parkinsonism.

Clinical data pertaining to each participant was extracted from their medical records. This information included age, gender, body weight, disease duration, age of onset, and the type of PD, which was categorized into tremor dominant, intermediate, and akinetic rigid types. At the point of PD diagnosis, we evaluated the initial motor severity during the off status using the Unified Parkinson’s Disease Rating Scale (UPDRS)-III, as well as the modified H&Y stage and the Korean version of the Mini-Mental State Examination (K-MMSE) to assess cognitive function. To quantify the levodopa treatment, we calculated the levodopa equivalent daily dose (LEDD) 3 months after the initial diagnosis, following a previously published method (Tomlinson et al., 2010). Furthermore, all PD participants went through brain magnetic resonance imaging (MRI) at the time of PD diagnosis to assess the severity of white matter changes using the modified Fazekas scale. This study was conducted with approval from the ethics committee of Gangneung Asan Hospital.

### Assessment of LID

The presence of LID was assessed through regular neurologic examinations and history taking at outpatient clinic visits every 2–3 months. LID assessments were conducted over a longitudinal follow-up period of at least 30 months after PD diagnosis by 1 movement disorder specialist (WJ).

### Assay for heavy metal levels

Whole blood samples were collected from all enrolled PD subjects during their hospitalization for PD diagnosis workup. After an overnight fasting period, blood sampling was performed in morning to ensure consistent conditions for analysis. Having packed with ice to maintain stability, samples were transmitted to Eone Laboratories Corporation (Incheon, South Korea) for heavy metal analysis. Zn, Cu, Pb, Hg, and Mn levels were assayed using inductively coupled plasma–mass spectrometry (ICP–MS) at Eone Laboratories. Serum was attained after centrifuging the whole blood sample at 200 x g for Zn and Cu measurements, and the analysis was performed using Agilent 7,900 ICP–MS. On the other hand, Hg, Mn, and Pb levels were measured using the whole blood sample with Agilent 7,700 ICP–MS. A 200-μL aliquot of the sample was diluted (1:10) with 1800 μL of 1% HNO3 and then centrifuged at 701 x g for 1 min before analysis. The assays were performed by pursing the standard guidelines from the manufacturer to ensure accuracy and reproducibility. By using distilled water, a mock blood draw was implemented to check any chance of elements contaminated from the collection tubes. The distilled water was collected and processed via same instructions and substances as that of the actual blood samples. Subsequently, the water samples were analyzed for the presence of heavy metals by ICP–MS. No significant contamination was found in distilled water samples, verifying that the collection tubes did not carry substantial levels of heavy metals into the blood samples. For quality control and assurance, standard reference materials for trace element serum and metals in whole blood from UTAK Laboratories Inc. were utilized for both Agilent 7,900 and 7,700 ICP–MS analyses. The interday and intraday coefficients of variation of all tests were found to be below 10%, ensuring the reliability and precision of the heavy metal measurements.

### Quantitative analysis of FP- CIT PET/CT

FP-CIT PET/CT was performed in all subjects at baseline. PET/CT scans were obtained 2 h after an intravenous injection of an average of 185 MBq (5 mCi) of FP-CIT. All of the subjects underwent PET/CT imaging in a Discovery ST8 scanner (GE Healthcare). The emission PET data were acquired for 15 min, and the CT data were used for attenuation correction. The dopamine transporter binding state was evaluated through a semiquantitative analysis of FP-CIT PET/CT. The FP-CIT PET images were spatially normalized to MNI (Montreal Neurological Institute) space by using a standard FP-CIT PET template and Statistical Parametric Mapping 8 (SPM8) software implemented in MATLAB R2022b for Windows (The MathWorks Inc.) as previously described (Oh et al., 2012). Regional mean standardized uptake values (SUV_mean_) were extracted from both caudate nuclei and both the putamina and occipital cortex by using volume-of-interest templates drawn on the MNI space. Semiquantitative analyses were performed using the specific binding ratio (SBR) and the asymmetry index (AI) (Youn et al., 2018). The SBRs of the putamen and caudate nuclei were calculated as follows: (striatal SUV_mean_ – occipital SUV_mean_)/occipital SUV_mean_. The AIs of the putamen and caudate nucleus were calculated as follows: (higher striatal SBR – lower striatal SBR)/(higher striatal SBR + lower striatal SBR).

### Statistical analysis

Statistical analyses were carried out using GraphPad Prism 9.0 software (GraphPad Software, Inc., San Diego, CA). Categorical variables were compared using *χ*^2^ tests, while continuous variables were compared using independent *t* tests. Initially, a comparison was made between demographic and clinical parameters and heavy metal levels in PD patients with and without LID. Then, we adopted correlation analysis to reveal the relationship between heavy metal levels in significance and diverse clinical measures of PD, the age of onset, LEDD at 3 months, K-MMSE, UPDRS-III, and parameters from FP-CIT PET/CT. Cox proportional hazards regression analysis was done in order to analyze factors relevant to the time to LID development in PD subjects. A stepwise variable selection approach, combining forward and backward selection, was used to select the variables for the regression model. The Akaike information criterion was utilized to identify the best-fitting model. For all analyses, the significance value was *p* < 0.05.

## Results

### Demographic traits of PD subjects

Table 1 presents the demographic characteristics of the participants. PD patients with LID were younger than PD patients without LID, including their age of onset (70.63 ± 10.60 versus 75.65 ± 8.33 years, *p* < 0.01; 63.78 ± 10.21 versus 69.80 ± 8.38 years, *p* < 0.01). PD patients with LID also demonstrated more demanding clinical measures than non-LID patients, including the following: longer disease duration, higher UPDRS-III scores, and LEDD at 3 months (92.46 ± 19.82 versus 79.08 ± 28.24 months, *p* < 0.01; 27.80 ± 5.58 versus 24.26 ± 8.33, *p* = 0.01; 674.33 ± 166.93 mg versus 571.83 ± 168.36, *p* < 0.01). PD patients with LID demonstrated a higher AI of the putamen (1.63 ± 0.89 versus 1.25 ± 0.20, *p* < 0.01). Finally, PD patients with LID had a higher prevalence of the akinetic rigidity subtype than those without LID (*p* < 0.01).

**Table 1:** Comparison of basic demographic and clinical parameters and serum heavy metal levels between PD patients with LID and PD patients without LID.

	PD without LID (*n* = 65)	PD with LID (*n* = 46)	*p* value
Age (years)	75.65 ± 8.33	70.63 ± 10.60	<0.01
Gender (male/female)	29/36	20/26	0.90*
Weight (kg)	59.50 ± 11.01	64.11 ± 10.49	0.53
Disease duration (months)	79.08 ± 28.24	92.46 ± 19.82	<0.01
Type of PD (TD/intermediate/AR)	33/19/13	16/8/22	<0.01*
Age of onset (years)	69.80 ± 8.38	63.78 ± 10.21	<0.01
UPDRS-III	24.26 ± 8.33	27.80 ± 5.58	0.01
LEDD at 3 months	571.83 ± 168.36	674.33 ± 166.93	<0.01
Modified H&Y	1.94 ± 0.56	1.91 ± 0.54	0.80
K-MMSE	24.43 ± 4.21	24.83 ± 5.16	0.66
AI of putamen	1.25 ± 0.20	1.63 ± 0.89	<0.01
AI of caudate	1.23 ± 0.27	1.34 ± 0.80	0.27
SBR of putamen	1.66 ± 0.57	1.58 ± 0.80	0.51
SBR of caudate	1.60 ± 0.62	1.75 ± 0.87	0.31
Heavy metals			
Zinc (μg/dL)	88.16 ± 15.15	79.58 ± 12.28	<0.01
Copper (μg/dL)	101.09 ± 20.03	96.82 ± 22.00	0.29
Lead (μg/dL)	1.78 ± 0.74	1.85 ± 1.22	0.69
Mercury (μg/L)	2.85 ± 2.28	2.64 ± 1.92	0.62
Manganese (μg/L)	11.17 ± 3.35	10.47 ± 3.99	0.31

These values represent the mean and the standard deviation or the number of subjects. *Chi-squared test. PD, Parkinson’s disease; TD, tremor-dominant; AR, akinetic-rigidity; UPDRS-III, United Parkinson’s Disease Rating Scale Part 3; LEDD, L-DOPA equivalent daily dose; H&Y, Hoehn and Yahr stage; K-MMSE, Korean version of the Mini-Mental State Examination; AI, asymmetry index; SBR, specific binding ratio.

### Heavy metals and PD

**Figure 1:**
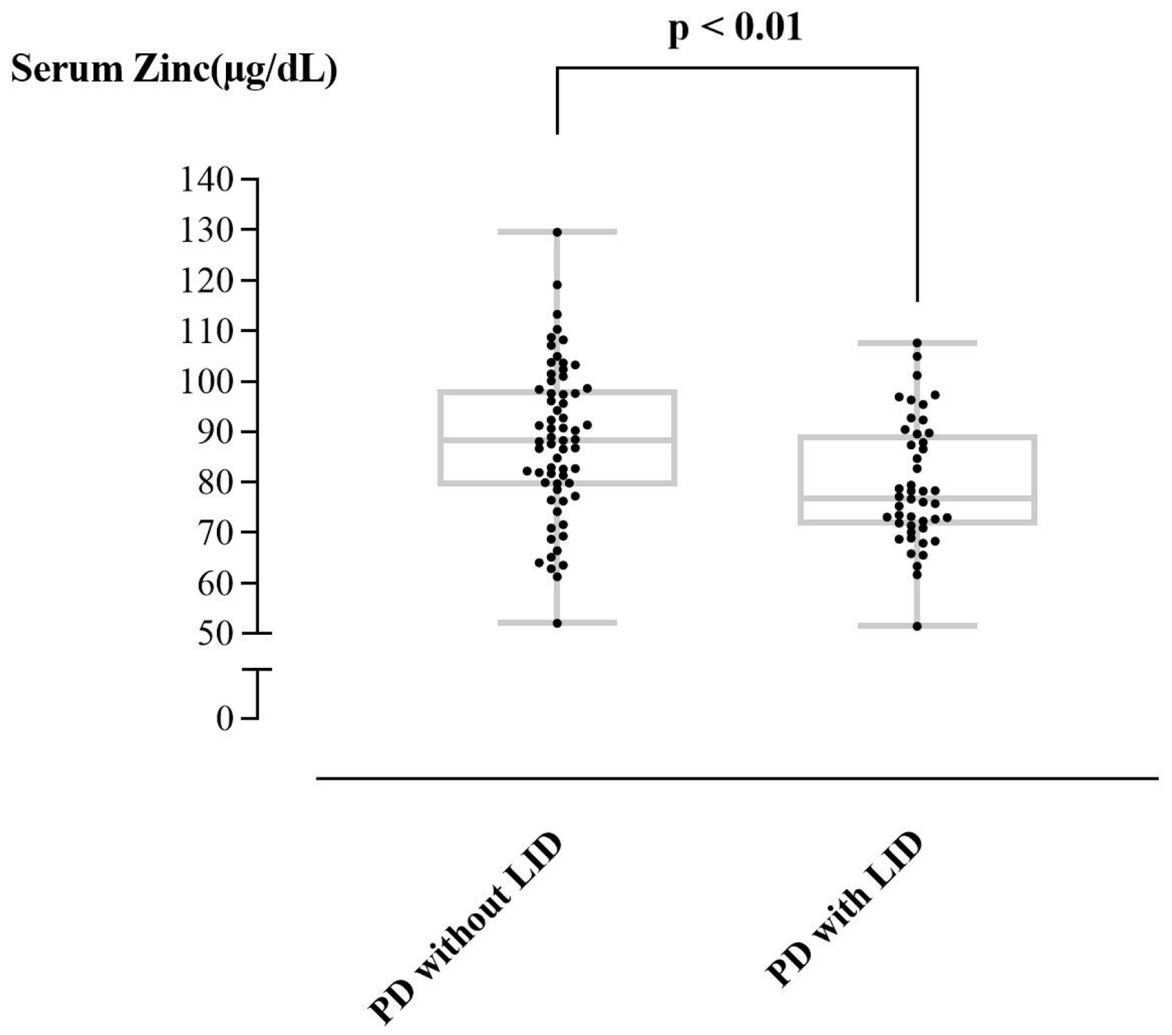
Comparison of serum Zn between PD patients with and without LID.

Table 1 shows heavy metal levels in PD patients with and without LID. PD patients with LID had lower levels of Zn, while Zn appeared to be the only heavy metal presenting a significant difference among the five heavy metals (79.58 ± 12.28 versus 88.16 ± 15.15 μg/L, *p* < 0.01; reference range: 81.0 ~ 121.0 μg/L). Figure 1 compares the Zn levels between PD patients with and without LID.

Table 2 shows correlation analysis between Zn level and clinical parameters of PD adjusting for age, revealing that Zn level was dominantly correlated with K-MMSE (*r* = 0.28, *p* < 0.01), followed by age of onset (*r* = 0.16, *p* < 0.05). LEDD at 3 months (*r* = − 0.20, *p* < 0.01) showed a strong negative correlation with statistical significance. Table 2 also shows correlation analysis between Cu level and clinical parameters of PD, with LEDD at 3 months (*r* = − 0.20, *p* < 0.05) also presenting negative correlation with statistical significance.

**Table 2:** Partial correlation analysis between clinical parameters and serum zinc, copper in PD patients.

	Serum zinc (μg/dL)		Serum copper (μg/dL)	
	*r*	*p*	*r*	*p*
Age of onset	0.16	<0.05^*^	−0.86	0.37
Weight (kg)	0.01	0.92	−0.11	0.27
UPDRS-III	−0.13	0.06	−0.04	0.70
LEDD at 3 months	−0.20	<0.01^**^	−0.20	<0.05^*^
K-MMSE	0.28	<0.01^**^	0.13	0.19
AI of putamen	−0.12	0.09	0.12	0.21
AI of caudate	−0.04	0.60	0.17	0.07
SBR of putamen	0.01	0.85	−0.01	0.97
SBR of caudate	−0.02	0.79	−0.04	0.69

*r*, Pearson correlation coefficient. *p < 0.05, **p < 0.01.

PD, Parkinson’s disease; LEDD, L-DOPA equivalent daily dose; UPDRS-III, United Parkinson’s Disease Rating Scale Part 3; K-MMSE, Korean version of the Mini-Mental State Examination; AI, asymmetry index; SBR, specific binding ratio.

### Predictive factors for time to LID conversion in PD patients

The univariate model demonstrated that the type of PD was in relation with LID conversion in PD subjects (HR 0.398, 95% CI 0.207–0.765, *p* < 0.01; HR 0.418, 95% CI 0.186–0.943, *p* < 0.05, respectively, Table 3). Other clinical factors, such as weight, LEDD at 3 months, and UPDRS-III, were also associated with time to LID conversion and AI of the putamen (HR 1.036, 95% CI 1.009–1.064, *p* < 0.05; HR 1.003, 95% CI 1.001–1.005, *p* < 0.01; HR 1.073, 95%, CI 1.026–1.123, *p* < 0.01; and HR 1.381, 95% CI 1.056–1.805; *p* < 0.05, respectively). Among heavy metals, Zn and Cu levels were also associated (HR 0.967, 95% CI 0.946–0.988, *p* < 0.01; HR 0.982, 95% CI 0.967–0.997, *p* < 0.05).

**Table 3:** Univariate and multivariate Cox proportional hazards regression analysis for predicting LID conversion in PD subjects.

Covariates	Univariable analysis				Multivariable analysis			
	HR	95% CI		*p* value	HR	95% CI		*p* value
		Lower	Upper			Lower	Upper	
Age of onset	0.974	0.945	1.005	0.09	0.942	0.910	0.975	<0.01
Weight (kg)	1.036	1.009	1.064	<0.05				
Sex								
Male	Ref							
Female	1.570	0.870	2.833	0.13				
Type								
AR	Ref							
TD	0.398	0.207	0.765	<0.01				
Intermediate	0.418	0.186	0.943	<0.05				
LEDD	1.003	1.001	1.005	<0.01	1.002	1.000	1.004	<0.05
UPDRS-III	1.073	1.026	1.123	<0.01	1.088	1.031	1.148	<0.01
AI of putamen	1.381	1.056	1.805	<0.05				
Zinc (μg/dL)	0.967	0.946	0.988	<0.01	0.978	0.956	0.999	<0.05
Copper (μg/dL)	0.982	0.967	0.997	<0.05				
Lead (μg/dL)	0.952	0.693	1.308	0.76				
Mercury (μg/L)	0.869	0.748	1.010	0.07				
Manganese (μg/L)	0.943	0.872	1.020	0.14				

HR, hazard ratio; CI, confidence interval; AR, akinetic-rigidity; TD, tremor-dominant; LEDD, L-DOPA equivalent daily dose; UPDRS-III, United Parkinson’s Disease Rating Scale Part 3; AI, asymmetry index.

**Figure 2:**
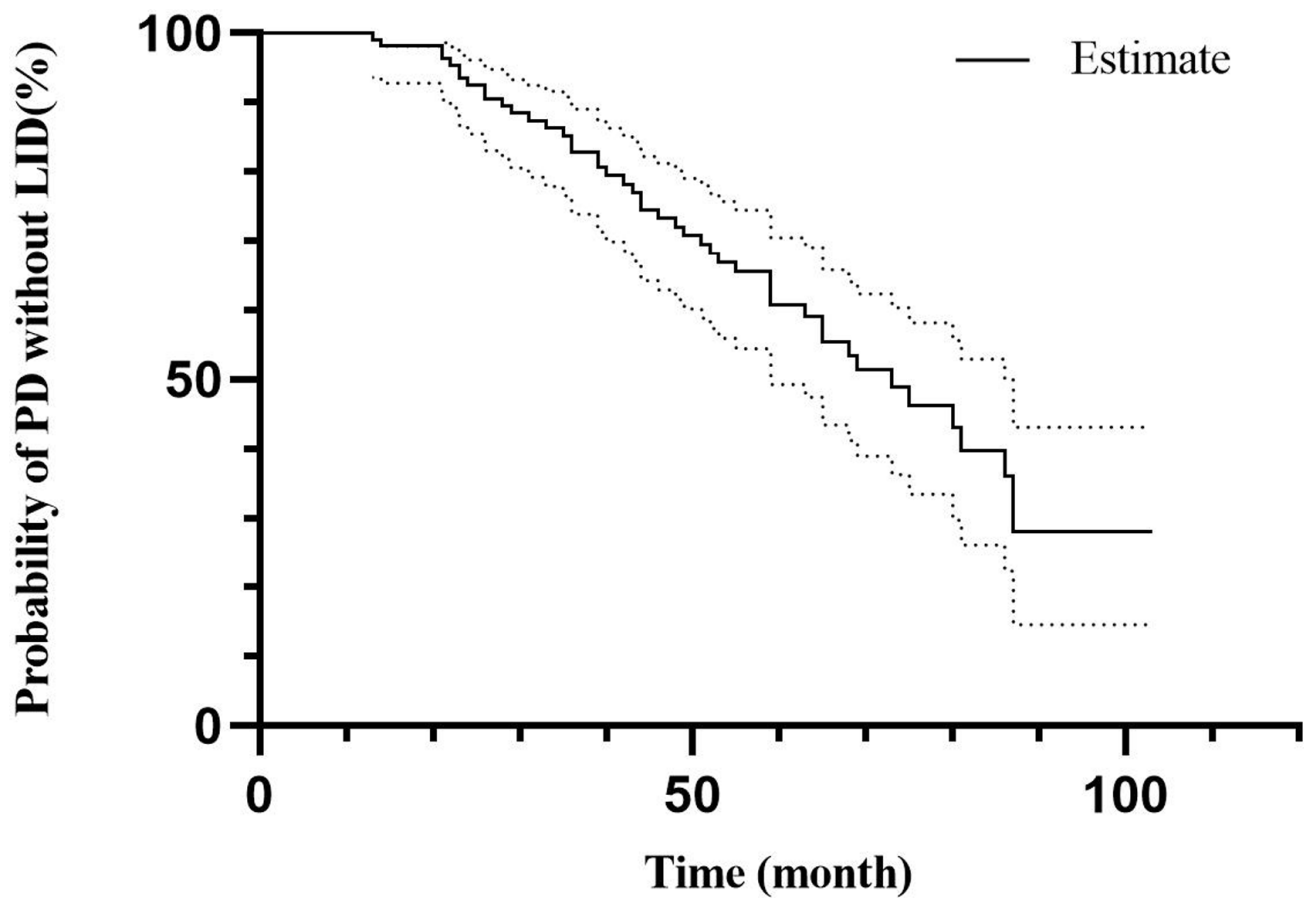
Risk of LID conversion in PD patients.

The multivariate model chose age of onset, LEDD at 3 months, UPDRS-III, and serum Zn level for predicting LID conversion in PD patients (HR 0.942, 95% CI 0.910–0.975, *p* < 0.01; HR 1.002, 95% CI 1.000–1.004, *p* < 0.05; HR 1.088, 95% CI 1.031–1.148, *p* < 0.01; and HR 0.978, 95% CI 0.956–0.999, *p* < 0.05 respectively, Table 3). Figure 2 depicts the probability of PD without LID according to time.

## Discussion

This study yielded two important findings. First, serum Zn levels in the LID group were significantly reduced compared with those in the PD without LID group. Furthermore, serum Zn levels showed a significant correlation at the diagnosis point, and presenting a significant correlation as well with age of onset, LEDD at 3 months, and K-MMSE scores. Second, Zn deficiency affected to a shorter time to LID development in the adjusted model. To the best of our knowledge, this is the first study revealing the possible longitudinal influence of Zn deficiency on LID development in PD.

Although it remains unclear, a number of epidemiological studies have depicted a significant association between PD and long-term exposure to heavy metals such as Pb, Hg, Cu, Mn, Aluminum(Al), and Zn (Fukushima et al., 2010; Zhao et al., 2013; Kim et al., 2018; Yoo et al., 2018). The central source of metal-associated PD incorporates occupational exposure, pollution, medication, contaminated seafood, and restorations of dental metals such as amalgam fillings (Di Monte et al., 2000; Raj et al., 2021). One study showed that exposure to heavy metals such as Fe, Mn, and Al doubled the risk of PD (Hong et al., 2014). Another study showed that an individual with exposure to Pb, Cu, and Mn for more than 2 years had a 2–10 times enhanced chance of PD (Atarod et al., 2022). Regarding Zn, although there are conflicting results, many studies have shown that Zn could be a risk factor for PD (Du et al., 2017; Sun et al., 2017; Krall et al., 2020; Sikora et al., 2020).

It has been well investigated that a number of metals penetrate the blood–brain barrier (BBB) (Xie et al., 2023). They could be accumulated in human brain, and affect the central nervous system(CNS) (Civit et al., 2000; Grochowski et al., 2019). But whether heavy metal level change is the triggering factor or the consequence of the CNS neurodegeneration remains vague. For instance, Fe could enter the brain either bound to transferrin or unbound form crossing BBB. Fe overloading could cause neurodegeneration through various mechanisms including apoptosis, autophagy, and ferroptosis (Dixon et al., 2012). However, the alternate perspective should be considered, which posits that changes in Fe levels are mere a consequence of neurodegeneration. Many evidence showed that Fe accumulation in the substantia nigra of PD was associated with motor severity and disease progression. In this perspective, heavy metal deficiency is more likely to be a consequence of CNS degeneration (Mochizuki et al., 2020). Du et al. (2022) also have demonstrated that nigral Fe was lower at the time of starting the dopaminergic medication, then had a tendency to increase until it reached the plateau at late stages, which indicate increased nigral Fe might not be an etiological factor. Therefore, change of heavy metal level observed in PD patients may simply be a consequence of the disease progression, with no direct causal relationship to neurodegeneration itself. Elucidating the exact mechanisms and causal relationships among heavy metals level, neurodegeneration, and disease progression remains a complex challenge that necessitates further investigations.

In our study, Cu and Zn were significant predictive factors for LID development in the univariate model, which indicates that these two heavy metals could prompt the neurodegenerative process in PD. Furthermore, there was significant correlation between serum Cu level and LEDD in our study. Cu plays a role as a cofactor in many key enzymes, including cytochrome c oxidase, Cu/Zn superoxide dismutase, dopamine β-hydroxylase, and ceruloplasmin, and interrelate with the pathogenic α-synuclein (αSyn) protein to deter the formation of αSyn filaments with toxic αSyn oligomer aggregation (Forsleff et al., 1999; Brewer et al., 2010; Double, 2012; Zhao et al., 2013; Davies et al., 2016). Zhao et al. (2023) reported that serum concentrations of Cu and Fe were lower in PD patients and Genoud et al. (2020) suggested that Cu level in the substantia nigra were significantly reduced in a meta-analysis. Kim et al. (2018) revealed that in women with PD, higher serum Cu levels or lower serum Fe levels affected LID occurrence and that serum Cu levels were in negative correlation with MMSE scores in PD patients. However, our results revealed that low Cu levels could be associated with shortened LID development. Furthermore, the multivariate model excluded serum Cu levels in the final model. Therefore, the influence and mechanism of serum Cu levels on LID development remain inconclusive.

Meanwhile, the most important finding in our study is that Zn deficiency could be a potential risk factor for LID occurrence. This finding could be explained by the following. First, the acceleration of presynaptic nigrostriatal degeneration by Zn deficiency could influence the development of LID. Zn is an essential trace element and an indispensable functional component of many enzymes, which both animals and humans require (Szewczyk, 2013). This enzyme, which is highly concentrated in the substantia nigra, is a superoxide dismutase that catalyzes the redox alteration of superoxide anions to hydrogen peroxide and oxygen, rigorously finding free radicals, securing these neurons from oxidative stress (Sun et al., 2017; Bakulski et al., 2020) and reducing stress in the hippocampus and cerebral area. In PD, loss of the substantia nigra and oxidative stress have both been characterized; thus, decreased Zn levels can provoke oxidative stress as well as free radical elevation, which eventually can damage proteins and DNA (Orisakwe, 2014; Parmalee and Aschner, 2017). This implies that Zn deficiency contributes to the degeneration process of PD (Ball et al., 2019). Second, low serum Zn levels could be associated with a higher dose of L-DOPA, which is a strong indicative factor of LID development. Matsuyama et al. revealed that serum Zn levels were negatively associated with levodopa dosage and frequency (Matsuyama et al., 2021). Zn deficiency could be triggered by inadequate Zn intake, insufficient absorption, and increased need and excretion, which refers to continuous use of chelating drugs such as levodopa (Tomita and Yoshikawa, 2002; Szewczyk, 2013; Matsuyama et al., 2021). Therefore, there is a possibility that the Zn-L-DOPA interaction could affect the total LEDD, which leads to a shortening of LID development. Yasuda et al. found that in Japanese adults, there is a highly significant inverse correlation between age and Zn concentration in hair (Yasuda and Tsutsui, 2016; Matsuyama et al., 2021), and with age covered as one of the variables in multiple regression analysis, a significant negative correlation in levodopa dosing frequency was found (Yasuda and Tsutsui, 2016). This implies that frequent levodopa administration affects PD patients’ serum Zn levels, regardless of age (Matsuyama et al., 2021). However, Zn levels were measured at the time when levodopa has never been administered in this study, and the length of the disease have been adjusted in statistical model. It is less likely that Zn levels affect the blood circulation directly. Third, Zn could influence glutaminergic neurons because of its contribution to LID. Depending on the synaptic targets, Zn ions within the striatum could perform either inhibitory or disinhibitory modulatory actions (Matsuyama et al., 2021; Sikora and Ouagazzal, 2021). One of the renowned distinct targets of synaptic Zn is N-methyl-D-aspartate receptors (NMDARs), with the upregulation and downregulation of the GluN2A and GluN2B subunits at the synaptic level (Krall et al., 2020). GluN2A-containing NMDARs are the major postsynaptic targets of synaptically released Zn due to their sensitivity to nanomolar concentrations of extracellular Zn, which inhibit receptor function (Krall et al., 2020). Considering that NMDA antagonists such as amantadine ameliorate LID severity, Zn deficiency could elicit a loss of modulatory function on NMDARs in the striatum and result in LID development (Thomas et al., 2004). Finally, Zn could also figure prominently in epigenetic modification. Zn is essential for the activity of various epigenetic enzymes, including DNA methyltransferases (DNMTs), histone acetyltransferases (HATs), histone deacetylases (HDACs), and histone demethylases, which have various Zn binding sites (Brito et al., 2020). Therefore, the disruption of Zn homeostasis could cause epigenetic process modification. Many studies have revealed that these epigenetic modifications of striatal neurons, especially sustained dopaminergic medication, have been suggested as one of the possible mechanisms for LID. Figge et al. (2016) suggested that modulation of DNA methylation in striatal neurons is required for LID development. Nicholas et al. (2008) also revealed that modulation of striatal histone modification could be a useful therapeutic mark for improving LID in PD patients. Therefore, if Zn might contribute to epigenetic processes, Zn deficiency could also be involved in LID pathogenesis.

Several limitations can be mentioned as following. First, due to its trait as a retrospective study, the observation gap of the patients varies. It is probable that patients have been censored due to involuntary movement progression, and PD with LID might have been distinguished more easily in patients who had regular clinical check-ups. Both cases would have affected the over- and underestimation of PD with LID. Second, numerous former studies on the connection between heavy metal exposure and PD risk depicted a correlation of chronic exposure and PD risk. However, in this study only the serum level was evaluated at diagnosis cross-sectionally. Therefore, exposure or source duration was not taken into account in this study. Third, metals were only assessed from serum, not cerebrospinal fluid (CSF) nor brain tissue. Although relationship between serum and brain metal levels may be intricate, a biomarker running with blood tests could be more feasible than that of brain tissue (Kim et al., 2018). Fourth, the definite point of when LID occurred could be not found since the determination of LID onset was based on the medical chart review. Furthermore, we could not rule out potential confounding factors that could affect LID development, such as genetic and epigenetic factors. Also, as previously mentioned, among many types of heavy metal, this study focused on 5 types: Zn, Cu, Pb, Hg, and Mn. This does not signify any depreciation of other metal such as Fe. Fe is one of the renowned substance that piles up in the brain, with its increased level present especially in the nigrostriatal dopaminergic system in PD patients (Chen et al., 2019). Considering its importance in pathophysiology of PD, there is a need for a study that elucidates the relationship between the pathogenesis of PD and clinical symptoms and Fe levels in human-derived specimens such as serum or CSF. Finally, this study was conducted in a single center where most of the patients reside in rural areas and have a high chance of being influenced by a certain surrounding. In order to generalize the results, further nationwide multicenter studies are prerequisite.

Despite these limitations, this study confirmed an association between serum Zn levels and the duration of LID occurrence, suggesting that serum Zn deficiency might be a possible risk factor for a shorter time to LID development. Therefore, a further study is required to inspect the role of Zn in LID pathogenesis, by further chance leading to more effective treatment.

## Data availability statement

The raw data supporting the conclusions of this article will be made available by the authors, without undue reservation.

## Ethics statement

The studies involving humans were approved by Ethics committee of Gangneung Asan Hospital. The studies were conducted in accordance with the local legislation and institutional requirements. The ethics committee/institutional review board waived the requirement of written informed consent for participation from the participants or the participants’ legal guardians/next of kin because This is retrospective study and admitted by ethic commitee of Gangneung Asan Hospital as minimal risk study.

## Author contributions

JK: Formal analysis, Investigation, Writing – original draft, Visualization. HL: Data curation, Formal analysis, Methodology, Writing – review & editing. WJ: Formal analysis, Investigation, Writing – original draft, Conceptualization, Data curation, Funding acquisition, Methodology, Supervision, Validation.
